# Military applications of transcranial direct current stimulation (tDCS) for enhanced multitasking performance

**DOI:** 10.1186/s41235-025-00679-6

**Published:** 2025-10-19

**Authors:** Sydni M. Nadler, Holly A. Taylor, Tad T. Brunyé, Marissa Marko Lee, Sara Anne Goring, Nathan Ward

**Affiliations:** 1 Department of Psychology, Tufts University, Medford, MA USA; 2 Center for Applied Brain and Cognitive Sciences, Tufts University, Medford, MA USA; 3 U.S. Army DEVCOM Soldier Center, Natick, MA USA; 4 Developing Brain Institute, Children’s National Hospital, Washington, DC USA

**Keywords:** Transcranial direct current stimulation (tDCS), Multitasking, Dual-tasking, Task-switching

## Abstract

Effective multitasking in high-stakes military environments is critical yet often compromised by cognitive overload, leading to operational errors. This scoping review explores the potential of transcranial direct current stimulation (tDCS) as a cognitive enhancement tool for improving multitasking performance, with a focus on task-switching and dual-task paradigms. Evidence suggests that tDCS targeting the dorsolateral prefrontal cortex (DLPFC) shows promise in mitigating task-switching deficits and reducing dual-task interference, particularly under unpredictable or high-demand conditions. However, variability in outcomes, influenced by stimulation parameters, task characteristics, and individual differences, highlights the need for further refinement of this approach. The limited but emerging evidence on high-definition tDCS (HD-tDCS) is also discussed, emphasizing its potential for more precise targeting, though current findings show mixed efficacy for multitasking enhancement. Practical applications of tDCS for military training and operations are examined, including skill acquisition, analyst performance, and drone piloting, where optimized multitasking capabilities could alleviate cognitive overload and enhance operational efficiency. While the findings are encouraging, additional research is essential to establish standardized protocols and assess the real-world utility of tDCS in complex military scenarios. This review highlights the importance of advancing neuromodulation techniques to address the increasing cognitive demands of modern military operations.

## Significance statement

Modern military operations demand high levels of multitasking, where failures due to cognitive overload may have severe consequences. This review investigates transcranial direct current stimulation (tDCS) as a tool for enhancing task-switching and dual-tasking performance, two critical elements of multitasking. Findings highlight the potential for tDCS to mitigate cognitive bottlenecks, reduce task interference, and improve vigilance, offering promise for military training and the growing complexity of human-machine interactions in combat environments. While this review highlights encouraging evidence, it also identifies areas for refinement, underscoring the need for further research to standardize stimulation protocols and understand the role individual variability and task requirements play in tDCS outcomes. This work connects the neural underpinnings of multitasking with practical enhancement solutions, contributing to efforts to optimize human performance in challenging environments.

In high-stakes military contexts, multitasking can lead to critical errors due to cognitive overload. For instance, tank gunners are often required to monitor their surroundings, identify targets, and operate robotic controls simultaneously, which increases the risk of missing vital threats (i.e., errors of omission) or making dangerous mistakes (i.e., errors of commission). Similarly, air traffic control operators must monitor numerous aircraft while coordinating flight paths. As a result, they can experience "change blindness," failing to notice significant updates in dynamic scenarios due to attentional overload (Chérif et al., 2018). Addressing these challenges will require innovative approaches at the level of individuals, teams, and systems to ensure that critical information can be processed, comprehended, and acted upon under demanding multitasking conditions. This becomes increasingly important in the context of the overwhelming information requirements likely to be demanded by next-generation human–machine integrated formations. Cognitive enhancement research may be one way to realize this goal, helping to identify novel solutions to improve multitasking performance by optimizing the balance between task demands and the available resources for accomplishing a task.

Cognitive enhancement, defined as a subsection of psychological science intending to improve the learning, memory, and attention capabilities of healthy individuals (Juengst, 1998), has captured global attention. As such, it should be no surprise that cognitive enhancement and its associated techniques have emerged as notable topics of interest for military entities (Brunyé et al., 2024; Sheftick, 2018). Military interest in cognitive enhancement is grounded in optimizing and exceeding human cognitive capabilities to engage with the variety of complex tasks military personnel encounter now, and the anticipated future increases in these demands. The complex and dynamic nature of modern conflict demands superior decision-making and adaptability, and the job responsibilities that occupy daily military life require shifting and dividing attention across multiple tasks. The need for military personnel to balance multiple duties, often simultaneously, and at optimal levels draws particular attention to the pursuit of tactics to augment *multitasking* abilities.

In this review, we discuss the potential effectiveness of one cognitive enhancement technique, transcranial direct current stimulation (tDCS). tDCS is a common neuroscience tool used to noninvasively influence brain activity and has been used to enhance multitasking performance on cognitive tasks. Specific focus is given to task-switching and dual-task paradigms. Our goal is to evaluate whether tDCS could be a viable tool for improving multitasking in current and future military applications, while considering the importance of task demands, stimulation parameters, and individual differences.

## What does it mean to multitask?

Multitasking, broadly defined as the temporal overlap in cognitive processes involved in more than one task (Koch et al., 2018), is associated with various cognitive functions including executive control (also known as executive function or cognitive control), working memory, and attention (Bühner et al., 2006; Himi et al., 2019; Miyake et al., 2000). Executive control allows the individual to regulate their behavior, thoughts, and emotions to achieve goals (especially in complex or novel situations where routinized responses are insufficient), and monitor self-performance to allow behavioral adjustments based on rule changes. These processes are crucial for adaptive, goal-directed behavior. Working memory is a set of cognitive functions allowing individuals to temporarily maintain, process, and manipulate information. These processes are thought to underlie diverse higher-level cognitive functions such as problem-solving, decision-making, and language comprehension. Attention is a cognitive process that allows individuals to selectively focus on tasks and stimuli while ignoring goal-irrelevant information.

**Fig. 1:**
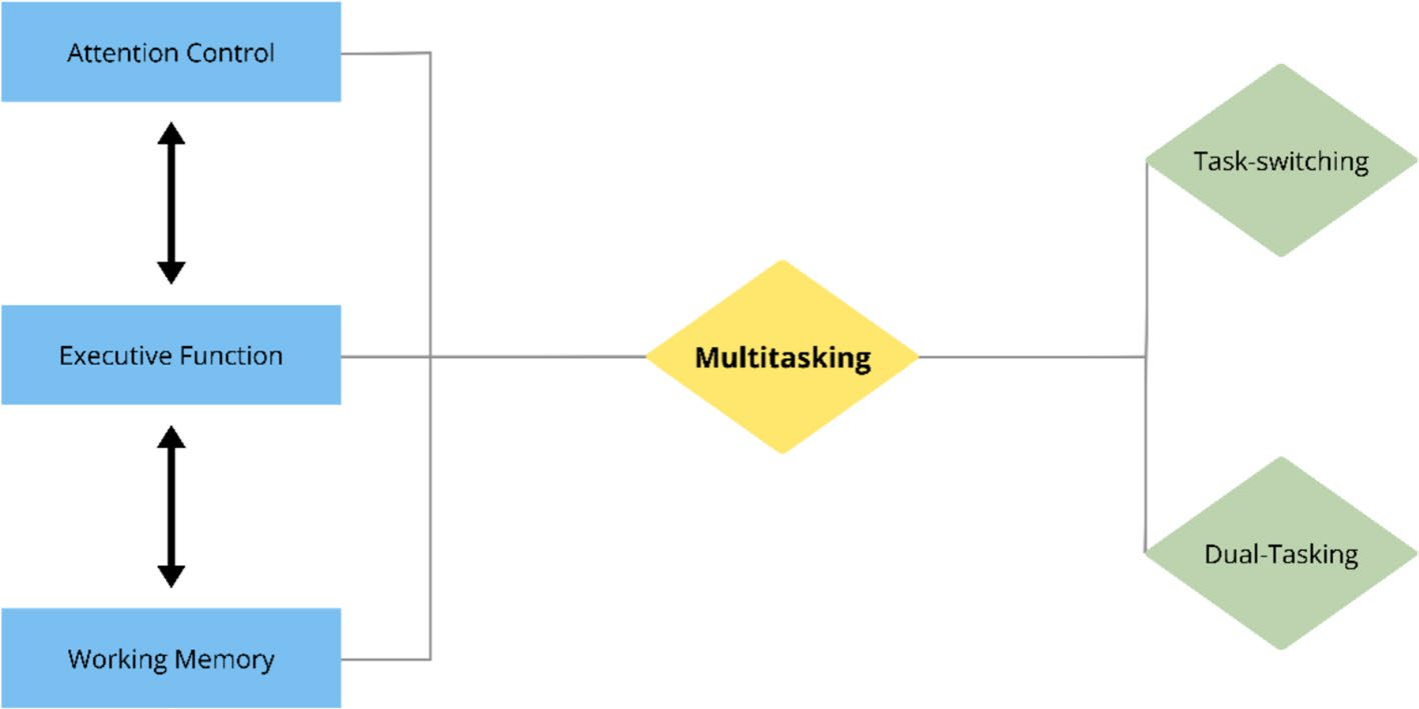
Contributing cognitive processes of multitasking and resulting paradigms

The brain networks involved in executive control, working memory, and attention primarily include the frontoparietal network (top-down regulation, task execution, and working memory manipulation), the cingulo-opercular network (sustained control and performance monitoring), and the dorsal and ventral attention networks (goal-directed focus and bottom-up attention to salient stimuli) (Corbetta & Shulman, 2002; Petersen & Posner, 2012). Subcortical structures like the basal ganglia and thalamus integrate and filter information (Himelstein et al., 2000), while the default mode network dynamically disengages during externally focused, cognitively demanding tasks (Christoff et al., 2016). Given the diverse ways in which people attempt to engage with multiple tasks at once and the array of cognitive processes and brain networks involved, two distinct approaches have emerged in the study of this essential ability: *task-switching* and *dual-tasking* (See Fig. 1).

Task-switching refers to the process of shifting attention between tasks and reconfiguring cognitive resources to accommodate multiple tasks (Monsell, 2003). Military and nonmilitary professionals task-switch constantly, such as having multiple tabs pulled up on a computer screen to enable alternating between responding to emails and drafting a work project. Task-switching is often a professional, if not personal, necessity, but not without limitations. The additional cognitive load required to alternate between tasks often results in a slower response or more errors, compromising overall task performance (Monsell, 2003). In a civilian scenario, this may lead to delays in sending an email or more typos in a project draft, but far more critical, even dangerous, issues could arise from switch costs in military settings (e.g., Loukopoulos et al., 2016). For example, Air Force officers may be required to monitor airspace radars and log flight information, going back and forth between visual vigilance and written communication. As temporal or computational demands increase, requiring faster switching or increased working memory burden, response latencies and errors are also likely to increase. Consequently, minimizing this effect while maximizing task-switching ability is a desirable outcome.

Dual-tasking involves an individual attempting to perform more than one task simultaneously without alternating between the two tasks. For example, infantry personnel may be visually scanning a scene while both listening and responding to orders over a radio. Civilian adults may dual-task by talking on the phone while drafting that aforementioned work project. Both activities require cognitive engagement and the successful application of executive control, working memory, and attention to be done successfully; when they are attempted simultaneously, success tends to be a challenge. The concurrent demand for shared cognitive resources between tasks, primarily attributed to processing bottlenecks (the point where overload occurs and subsequently limits the amount of information that may be simultaneously processed), ultimately leads to performance decrements in both response time and accuracy (Pashler, 1994). This is especially the case when one or both tasks become increasingly demanding. Improving dual-task abilities can be achieved through training (Dux et al., 2009; Shadrick & Lussier, 2004), but the drive to identify innovative solutions for mitigating dual-tasking impairments remains strong.

## Transcranial direct current stimulation as an enhancement technique?

tDCS is a form of noninvasive brain stimulation (NIBS) in which a weak electrical current applied directly to the scalp’s surface via electrodes can result in modifications of the underlying brain tissue’s neural activity. Modulation of the neural activity is thought to be dependent upon stimulation polarity: anodal stimulation can *depolarize* the cells, which encourages neuronal firing, whereas cathodal stimulation can *hyperpolarize* the cells, leading to inhibition of neuronal firing (Nitsche & Paulus, 2000, 2001). The target electrode, either anodal or cathodal, is placed over the cortical region of interest, while the reference electrode may be placed over another region of the brain or in extracephalic locations (often on the cheek or arm of the participant) to modify the flow of the tDCS current in the brain.

The efficacy of this technique, however, depends on a variety of factors; among others, variations in electrode location, strength of current intensity (usually in milliamps, mA), stimulation duration and timing (i.e., during a task, called *online* stimulation, versus before a task, called *offline* stimulation), and individual differences in participant samples (e.g., brain anatomy, initial brain state; Evans et al., 2022; Krause & Cohen Kadosh, 2014) each play roles. Given this extensive list of contributing factors, control conditions are essential when assessing the effects of tDCS. “Sham” stimulation, in which electrical current is ramped up to allow participants to feel common cutaneous sensations associated with stimulation, but then immediately ramped down to prevent any neural or behavioral modulation, is the most common control condition for this technique (Gandiga et al., 2006). Other prominent control conditions include ramping up at the beginning of a stimulation period and ramping down at the end to moderate recency effects and “active” controls, in which a cortical region irrelevant to a behavior of interest is stimulated (Woods et al., 2016). tDCS has gained popularity for its well-regarded safety profile (Grossman et al., 2019) and apparent myriad effects on cognitive functions, including multitasking.

These tDCS-induced multitasking effects, however, are not discovered randomly; they result from intentionally chosen electrode placements designed to target this cognitive skill and its component processes. For instance, both task-switching and dual-tasking have been linked to heightened activation in the prefrontal cortex, specifically the *dorsolateral* prefrontal cortex (DLPFC; Koechlin et al., 1999) and the inferior frontal junction (IFJ; Brass et al., 2005; Derrfuss et al., 2005); both of which are implicated in the frontoparietal network (Assem et al., 2020; Petersen & Posner, 2012). Studies targeting either brain region with tDCS may place electrodes over the left (F3) or right (F4) DLPFC, or left (FC3) or right (FC4) frontocentral region, according to the international 10–5 or 10–20 electroencephalogram (EEG) system. Based in the dorsal attention network (Petersen & Posner, 2012), the left and right posterior parietal cortex (PPC; P3; P4) are also common locations of interest in tDCS studies for their roles in multitasking (Dove et al., 2000; Sohn et al., 2000; Szameitat et al., 2002). As we will outline, these regions are chosen intentionally and appear repeatedly in research using tDCS to target multitasking performance; locations that deviate from these common areas are also tested (though less frequently) for their potential in modifying multitasking ability.

tDCS has been the focus of numerous reviews addressing its enhancing capabilities and its use in applied contexts. These include investigations into its use for multitasking (see Dedoncker et al., 2016; Strobach & Antonenko, 2017), military cognitive performance (see Brunyé et al., 2020, 2024; D’Alessandro et al., 2023; Feltman et al., 2020), and various applied settings where cognitive enhancement is critical (see Brunyé et al., 2019; Marois & Lafond, 2022). However, despite these comprehensive examinations, no reviews have specifically explored the intersection of these areas by examining tDCS as a tool to enhance multitasking ability within a military context. Multitasking is essential to military operations (Chérif et al., 2018), and as these demands grow (for example, with human–machine integration technology), a review of tDCS as a potential tool for enhancing multitasking ability in this setting is warranted.

## Scope of the present review: literature

To ensure we focus on the most relevant findings for military-inspired research, specific inclusion criteria were established to guide the article selection process. These criteria were designed to narrow the scope to studies that align with current advancements and address key factors influencing multitasking performance. This review is restricted specifically to peer-reviewed publications on tDCS and cognitive multitasking. Papers had to explicitly and *intentionally* assess task-switching or dual-tasking performance; those that *incidentally* analyzed variables associated with either multitasking type were not included in this review. We also excluded papers focused on cognitive-motor, rather than strictly cognitive, multitasking. Cognitive-motor multitasking is undoubtedly important in both military and civilian contexts (for example, see Mark et al., 2023). However, the selected focus was chosen to address the diverse nature of military duties beyond field operations, which often demand efficient and effective cognitive multitasking. Biomedical and pharmacological interventions administered alongside tDCS were excluded, as well.

In striving to adhere to the principle of use-inspired basic research, considerations were also given to the participant samples comprising the tDCS and cognitive multitasking literature. The US military maintains strict age (10 U.S.C. § 505) and health standards (*DoD Instruction 6130.03.*, 2024) for its enlisted members, and articles were included only if the participant sample was of healthy, neurotypical adults within enlistment age. It is important to note that certain neurological conditions (for example, attention deficit/hyperactivity disorder) do not universally prohibit military service. However, significant differences in tDCS outcomes between neurodivergent and neurotypical samples (see Berryhill & Martin, 2018; Turkeltaub et al., 2012) were determined to be confounding factors when assessing tDCS in the context of multitasking for the purposes of this review. Papers evaluating the effects of tDCS on clinical or neurodivergent populations were therefore excluded.

**Fig. 2:**
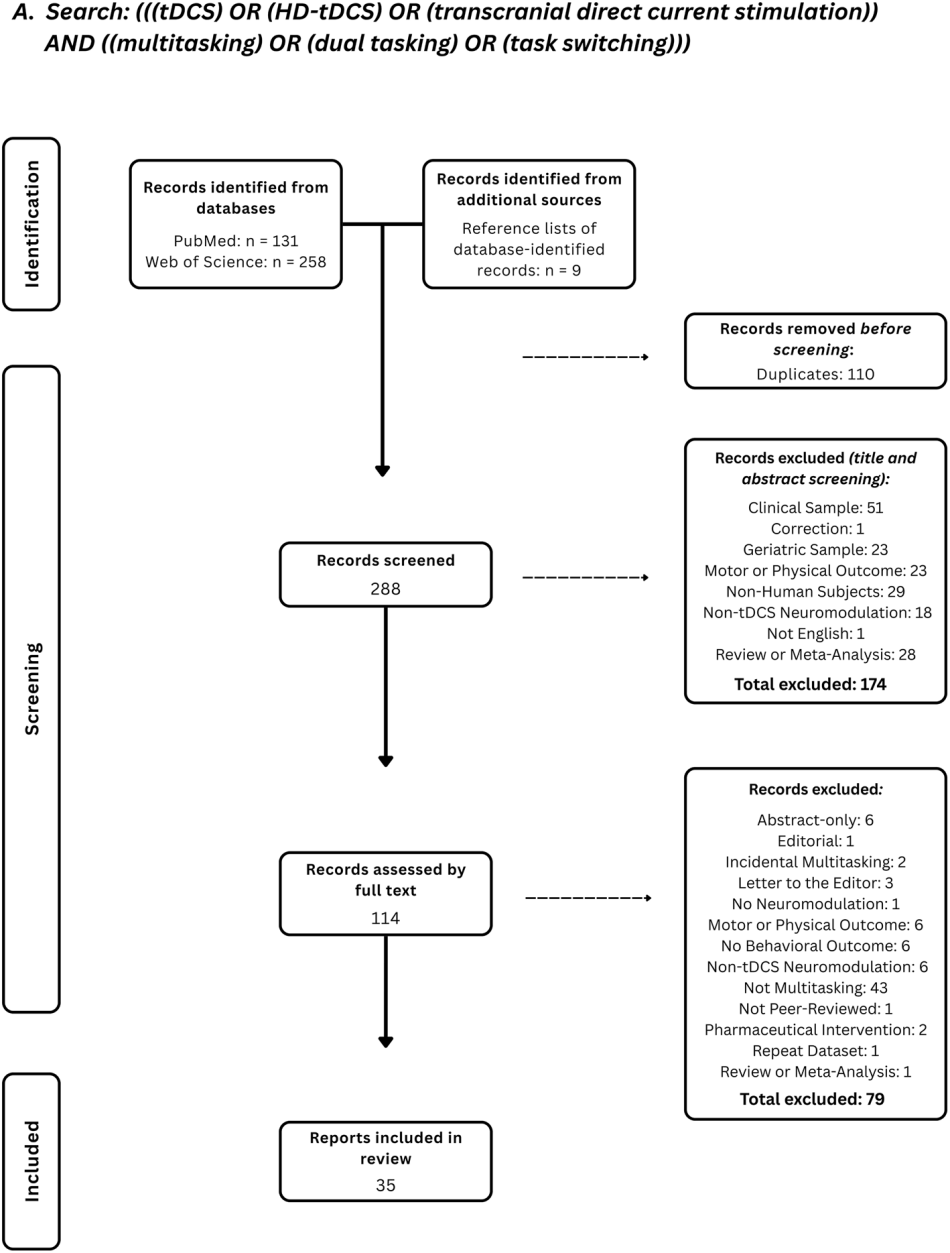

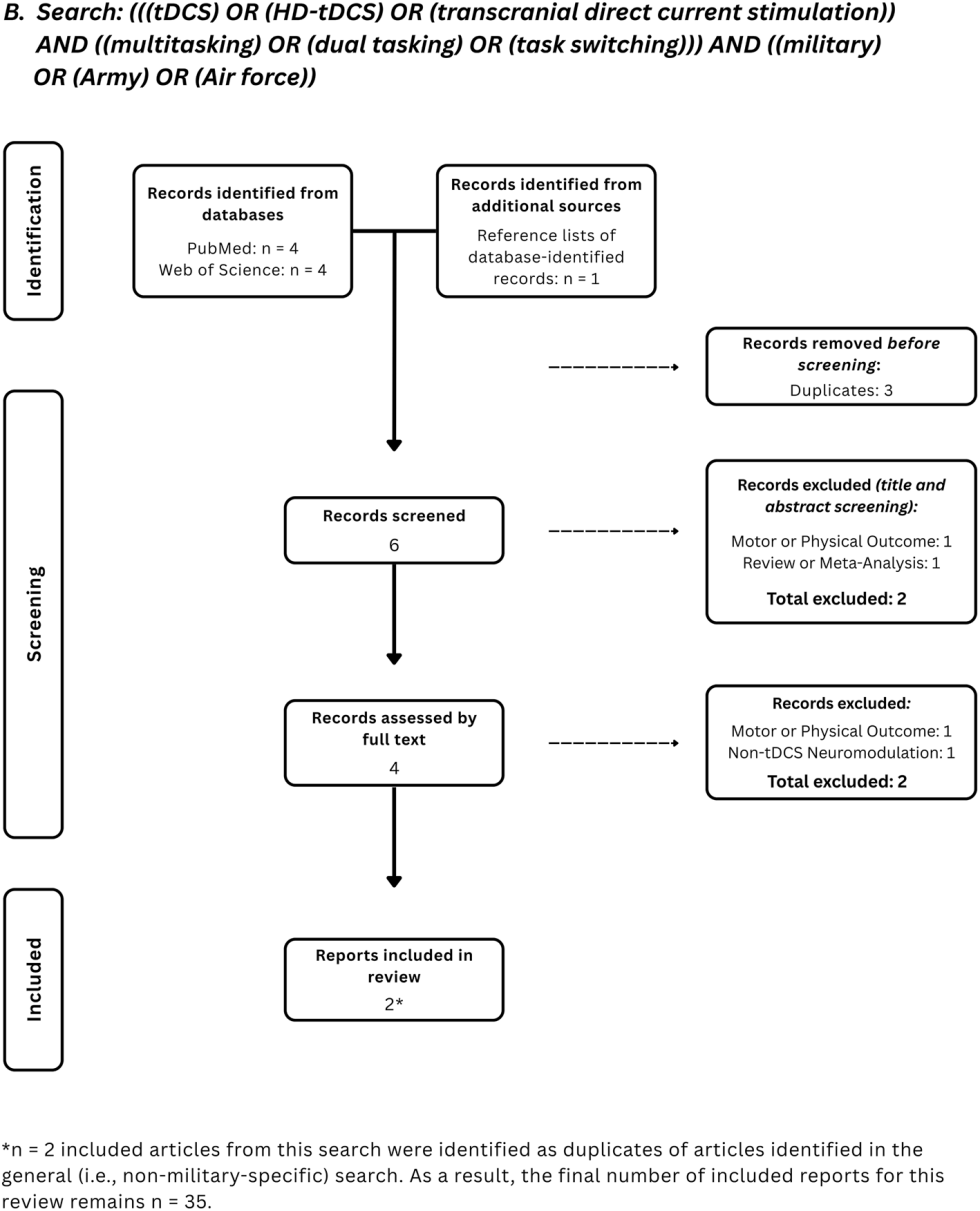
PRISMA flow diagrams of both general and military-specific records searches

Articles were initially sourced from Google Scholar, PubMed, and Web of Science in September 2021. Updated sourcing occurred between September and November 2024, and again in July 2025. The following operators were used in our searches: (“tDCS” OR “transcranial direct current stimulation” OR “HD-tDCS”) AND “multitasking” OR “dual task(ing)” OR “task switching” (See Fig. 2A). Searches focused on the military premise of this review were conducted using the combinations just specified AND “military” OR “Army” OR “Air Force” (See Fig. 2B). The references listed in each article identified through these searches were also used to source papers. We identified 35 articles that met these requirements.

A Risk of Bias assessment, using a modified version of the Downs & Black checklist (1998), was independently conducted by two authors (SMN, NW) to assess the methodological quality of these articles (See Appendix A for the checklist used, including explanations of modifications). This tool was selected for its ability to evaluate both randomized and unrandomized studies, as well as studies both with and without control conditions. Article quality was scored according to the percentage of checklist items reported out of 25 possible points. On average, studies included in this review scored 87.7% (Med = 88%; Range = 68%−96%) with high inter-rater reliability (*κ* = 0.901). As a result, these articles were considered to have considerable methodological quality.

## Task-switching

### Switch costs

As alluded to, the increased mental effort required to switch between tasks (e.g., many tabs and emails open on a computer screen) often contributes to impaired performance in those tasks. One form of impairment is referred to as a *switch cost*—the additional time and mental effort needed to disengage from one task and reconfigure cognitive processes to engage in another on a trial-level basis. In the context of task-switching, these costs typically result in slower reaction times and higher error rates on switch trials compared to repeat trials (Koch et al., 2018; Wylie & Allport, 2000). To reduce switch costs, several studies have explored the effectiveness of tDCS with mixed success. Thirteen such studies are evaluated here.

Across a variety of stimulation locations, tDCS has made little to no difference in switch costs, calling into question the use of this technology to improve task-switching ability. For example, when participants were asked to switch between categorizing a letter as a consonant or vowel and a number as odd or even, 20 min of 1.0 mA anodal stimulation over the left frontocentral region (between F3 and C3) did not contribute to changes in switch costs compared to a sham condition (Strobach et al., 2016). Nonsignificant effects of tDCS on switch effects have also been noted with 20 min of 2.0 mA stimulation targeting the left lateral frontal pole (F9) during a cued cognitive control task (Zacharopoulos et al., 2020). This, however, is not a trend isolated to the frontal lobe; similar nonsignificant findings have been noted with parietal stimulation. Following anodal stimulation to the right PPC (P4) Wang and colleagues (2024; 1.5 mA for 20 min) did not observe altered switch costs in the parity/magnitude task administered to their participants. Similar findings were also noted by Dubravac & Meier (2020), as neither left PPC (P3; 1.0 mA for 20 min) nor right parieto-occipital (P6; 1.0 mA for 20 min) anodal stimulation during a picture/word recall task yielded changes in participant switch costs.

In contrast, other studies have noted positive, task-specific effects of tDCS on switch costs. For example, Sdoia et al. (2020) had participants perform three numerical judgment tasks: a parity task, where they identified whether a digit was odd or even; a magnitude task, where they judged if the digit was smaller or larger than five; and a position task, where they determined if the digit was central or peripheral along a number line. These tasks were presented in sequences like ABA (returning to a previously inhibited task after performing a different one) and CBA (switching to a non-inhibited task), which allowed researchers to assess the effects of task inhibition and task reconfiguration on reaction times and accuracy under different stimulation conditions. It was noted that 1.5 mA of anodal tDCS over the right DLPFC (F4) resulted in general improvements in task-switching performance, with faster reaction times for both ABA and CBA task sequences when compared to sham. Anodal tDCS over the right PPC (P4) improved performance *exclusively* for the ABA task sequence. These results suggest that the DLPFC is involved in task-switching performance overall, whereas the parietal cortex may specifically manage task inhibition, a key factor in task-switching. However, Wang and colleagues’ (2022) also targeted the right parietal cortex (P4) with 20 min of 1.5 mA anodal tDCS. Contrasting the findings of Sdoia et al. (2020), they found that predictable task sequence (e.g., ABA) switch costs were significantly *higher* for stimulation of the right parietal cortex compared to sham stimulation and stimulation of the left parietal cortex. These discrepancies highlight the variability, and, at times, contradictory nature of tDCS outcomes, a primary critique of this neuroenhancement technique; despite similar tasks, stimulation parameters, and participant ages, these studies identified contradictory outcomes. It may be of value for future studies to account for individual differences with linear mixed effects models, evaluating the participant as a random effect. tDCS is influenced by such person-to-person variations (Evans et al., 2022; Krause & Cohen Kadosh, 2014), and taking statistical account of these variations may provide greater clarity as to why similar studies occasionally produce conflicting results and under what circumstances tDCS may be most beneficial.

These findings suggest that task-switching in military contexts could potentially benefit from targeted tDCS interventions given the right DLPFC’s involvement in general task-switching performance and the parietal cortex’s role in task inhibition. However, the variability in tDCS effects across studies also highlights the importance of tailoring stimulation protocols to specific tasks and individual differences to maximize their utility in such contexts; the risk of unintentionally increasing switch costs may outweigh any potential benefits seen with parietal stimulation.

Further highlighting the task-dependent nature of tDCS effects on switch costs, Wang et al. (2020) used a cued parity/magnitude task, manipulating task predictability by alternating between parity and magnitude judgments in either a fixed or random sequence. They found that 20 min of 1.5 mA anodal stimulation of the right DLPFC, but not the left, significantly reduced switch costs for unpredictable task sequences compared to sham stimulation. This result was corroborated in a follow-up study (Wang et al., 2021), where 1.5 mA anodal stimulation of the right DLPFC for 20 min again lowered switch costs for the unpredictable parity/magnitude task, but not for a parity/vowel-consonant task, relative to sham. Leite et al. (2013) also demonstrated the nuanced role of tasks when evaluating switch costs modulated by tDCS over the prefrontal cortex. In a letter/digit naming task, anodal tDCS over left DLPFC (F3) improved switching performance RT but not accuracy, while anodal tDCS over the right DLPFC (F4) enhanced accuracy but not RT. Conversely, in a vowel-consonant/parity task, Leite et al. (2013) saw that left DLPFC (F3) stimulation increased accuracy but impaired RT switching performance, underscoring the task-specific (and potentially hemisphere-specific) effects of tDCS on task-switching measures. The same parameters (30 min of 2.0 mA stimulation) were used to produce this variety of results in Leite et al.’s (2013) two tasks. Collectively, these findings indicate that targeting the DLPFC with tDCS under conditions of uncertainty may hold some promise in mitigating switch costs, though task demands and stimulation parameters may significantly moderate tDCS efficacy.

Interestingly, it appears that anodal tDCS targeting task-switching performance may have a stronger effect on response times than on accuracy. For both switching *and* repeating trials, 20 min of 1.5 mA anodal stimulation of the DLPFC, particularly the left, has been observed to significantly reduce response times while not impacting task accuracy (Tayeb & Lavidor, 2016). Leite et al., (2011; 1.0 mA for 15 min) and Prehn et al., (2021; 1.0 mA for 20 min) also noted reduced RT switch costs without effects on accuracy following anodal tDCS over DLPFC, respectively, assessing the left and right hemispheres. Compared with sham, though, 30 min of 2.0 mA stimulation to the right PPC (P4), rather than the DLPFC, *increased* response times of switch costs while leaving accuracy untouched (Leite et al., 2020). These findings collectively suggest that tDCS of the DLPFC may facilitate more efficient task-switching, but in the context of switch costs more generally, the ability to modulate switching performance with tDCS is far from uniform across tasks and conditions. Variations in stimulation parameters, electrode placements, and fluctuating task demands all contribute to the range of switch cost results noted in this section. Additional research is needed to further characterize the impact of tDCS on switch costs in the context of multiple manipulated and/or extraneous variables.

### Mixing costs

In addition to switch costs, task-switching paradigms often examine *mixing costs*, a critical measure used to evaluate the sustained cognitive demands of maintaining readiness for multiple tasks. Unlike switch costs, which reflect the immediate cost of transitioning from one task to another, mixing costs are calculated by comparing performance on repeat trials in mixed-task blocks (i.e., where multiple tasks are alternated) with repeat trials in single-task blocks (i.e., where only one task is performed; Koch et al., 2018; Los, 1996). In other words, mixing costs capture the *global* effort required to prepare for and maintain multiple task sets, rather than the *local* cost of executing a switch between tasks. Reducing mixing costs, and in turn, increasing preparedness to manage multiple tasks at once, has been examined in the tDCS and task-switching literature. This effect, however, has been examined less than switch costs, and outcomes appear to be inconsistent; indeed, we only identified five articles discussing the impact of tDCS on mixing costs.

Anodal tDCS for 20 min over both left and right DLPFC (Wang et al., 2020, 2021) and parietal cortices (Wang et al., 2022, 2024) before a mixed block parity/magnitude task rendered no significant changes in mixing costs between tasks. Strobach and colleagues (2016) further observed 20 min of 1.0 mA of anodal tDCS over the left DLPFC *increased* mixing costs compared to cathodal tDCS and sham. These results, while limited, suggest that tDCS effects on global mixing costs may differ from effects on local switch costs. This could potentially reflect distinct neural mechanisms underlying sustained task-set maintenance versus task-set reconfiguration; global task preparation, unlike local task-switching, may be less amenable to modulation via tDCS. Even more concerning, from the perspective of application, is that when performance is amenable, it may even be impaired. However, these conclusions are dependent on both stimulation and task parameters, as well as the limited body of research exploring mixing costs. The tDCS multitasking enhancement literature stands to benefit from additional assessments of mixing costs and a greater consensus among studies on electrode placements, stimulation frequency, and duration of stimulation.

### Interim summary

Task-switching involves transitioning between two or more tasks and is often accompanied by switch costs, evidencing the time and effort required to reconfigure cognitive processes for a new task. Switch costs are an unfortunate byproduct of attempting to shift attention between multiple tasks and can negatively influence applied task performance. Studies exploring tDCS to mitigate switch costs have yielded mixed results, though targeted stimulation of the right DLPFC shows some promise in specific task contexts (e.g., Sdoia et al., 2020). As observed by Wang and colleagues (2020; 2021), these performance benefits are particularly notable under conditions of unpredictability. Stimulation of the left DLPFC, on the other hand, may contribute to reduced switch costs (e.g., Leite et al., 2011; Prehn et al., 2021; Tayeb & Lavidor, 2016) However, variability in outcomes across studies highlights the potential influence of several relatively underexamined variables such as task demands, stimulation parameters, and individual differences.

Mixing costs reflect the sustained effort of maintaining readiness for multiple tasks, and have received less attention in tDCS research. The limited evidence suggests global task preparation may be less responsive to tDCS, potentially reflecting distinct neural mechanisms from task reconfiguration; in some cases, tDCS may even *increase* mixing costs (e.g., Strobach et al., 2016), an unfortunate outcome in the context of military application. Further research is needed to clarify tDCS effects on task-switching performance, focusing on employing more consistent experimental protocols and examining mixing and switch costs together.

## Dual-tasking

### Dual-task costs

Dual-task multitasking traditionally compares performance between single-task and dual-task environments. In these comparisons, dual-task costs emerge as the performance decline observed when two tasks are performed simultaneously, relative to when each is completed individually. These costs are a central focus in the study of dual-tasking, as they provide insights into the limitations of cognitive processing and the mechanisms of task interference. By quantifying dual-task costs, such as increases in response latency or in error rates, researchers can investigate how attentional resources are divided, how task priorities are negotiated, and what factors contribute to the cognitive bottlenecks that hinder multitasking efficiency. As with task-switching, varying evidence has emerged to support the use of tDCS in reducing dual-task costs, with effects often dependent on stimulation parameters and task demands. Eleven such articles are outlined here.

Particularly strong suggestions about the effectiveness of tDCS in improving dual-task ability has stemmed from its application over the DLPFC. For example, Hsu et al. (2015) observed enhanced multitasking efficiency in their single-blind, crossover study exclusively in the group of participants who first received active stimulation and then sham (i.e., active-sham). Compared to sham-active and sham-sham groups, participants who first received 10 min of 1.0 mA stimulation to the left DLPFC (F3) experienced less interference (i.e., decreased multitasking costs and increased target discrimination ability) from the presence of an additional task during a 3-D driving game. Not only does the study support the crucial role the left DLPFC plays in dual-tasking but also indicates that anodal tDCS over this area may enhance cognitive adaptations in dual-tasking over time. The sustained dual-tasking benefits of anodal tDCS to the left DLPFC are further supported by Filmer et al. (2017), whose combined tDCS and training paradigm showed both long-term and transferable dual-task benefits. An audiovisual identification task was used as their dual-task training paradigm. Enhancements in single- and dual-tasking were observed as a result of 13 min of 0.7 mA anodal stimulation and multitasking training shortly after the end of training. Up to two weeks later, enhanced performance was observed in a visual search task for the anodal stimulation group, as compared to cathodal or sham stimulation groups. Hsu et al. (2015) and Filmer et al. (2017) indicated the effectiveness of left DLPFC anodal stimulation for dual-tasking both in the immediate aftermath of stimulation and in the long-term.

The Filmer et al. (2017) study also lends credence to the efficacy of combining tDCS with multitasking training to yield dual-tasking performance benefits. In a comparison of anodal tDCS over F3, anodal tDCS over F3 with cognitive training, and sham stimulation with cognitive training, Martin and colleagues (2013; 2.0 mA for 30 min) observed enhanced dual-tasking accuracy in the group receiving active stimulation and training compared to sham with training. At posttest, both the sham and active stimulation + training groups also outperformed the tDCS only group in choice reaction time, and, at follow-up (at least four weeks after posttest), the tDCS + training group displayed transfer effects relative to the tDCS only group (though these effects were limited). Extending these findings, Rao et al. (2024) identified that administering 15 min of 1.0 mA stimulation to F3 during cognitive training improved dual-tasking ability in two of four subtasks compared to sham. These gains, though, were limited in an untrained dual audiovisual N-Back task. This pattern of findings, like those of Filmer et al. (2017), emphasizes that training can play an integral role when attempting to improve multitasking ability. However, these benefits do not always emerge in a trained paradigm. In a between-subjects study, Wards et al. (2023) paired four days of multitasking training with 13 min of either 1.0 mA anodal stimulation to F3 or F4, 2.0 mA anodal stimulation to F3, or sham stimulation; following the intervention, tDCS at either location or frequency was observed to alter dual-tasking costs in the trained task. Instead, the key behavioral effect of stimulation emerged in transfer. Regardless of laterality, participants who received 1.0 mA of stimulation demonstrated faster reaction times on an untrained visual search task, particularly at larger set sizes (where task demands are higher), relative to sham. These improvements persisted at a one-month follow-up, whereas 2.0 mA stimulation and control training conditions did not yield reliable transfer benefits.

Notably though, anodal stimulation to the *right* DLPFC, rather than the left, may *not* produce enhanced multitasking capabilities as consistently. Following 20 min of 2.0 mA anodal tDCS over the right DLPFC, Perrotta et al., (2021, Experiment 2) did not observe significant performance differences on a Stroop/N-back dual-task compared to sham. This lack of effect may be attributed to the laterality of the stimulation provided but may also be explained by the high cognitive demands of the Stroop/N-back dual-task paradigm. Variability in stimulation location, protocol, and task requirements, as suggested throughout this review, play strong roles in the effectiveness of tDCS in enhancing multitasking. The conflicting results discussed here highlight the complexity of drawing definitive conclusions about the utility of tDCS for multitasking and further argues the need for more standardized methodologies.

Evidence also suggests that variations in both tDCS protocol and in participant sample may play a role in its effectiveness in decreasing dual-task costs. For example, the range of 0.5 mA to 4.0 mA of current intensity is considered safe and tolerable for human use (Ko, 2021), and the studies evaluated in this review range from 0.7 mA to 2.0 mA with varying degrees of success in improving multitasking ability. This point is adeptly made by Ehrhardt and colleagues (2021), who evaluated the efficacy of 20 min of 0.7, 1.0, and 2.0 mA anodal stimulation over the left DLPFC (F3). Dual-tasking performance was evaluated with an audiovisual decision-making task. When compared to sham stimulation, only participants who received 1.0 mA stimulation experienced improvements in accuracy during dual-task conditions and quicker response times during transfer single-task conditions. Similar benefits in dual-tasking were not observed for the 0.7 mA or 2.0 mA groups, though other studies have seen improvements with those intensities at the same electrode site (Filmer et al., 2017; Nelson et al., 2016). These nuances are further highlighted by Martin and colleagues (2014; 2.0 mA for 30 min), who compared the effects of online and offline anodal tDCS to F3 on dual-tasking performance. Using a dual n-back cognitive training paradigm, they found that *online* stimulation produced superior dual-tasking skill acquisition during the initial task as well as sustained performance advantages the following day, whereas *offline* stimulation yielded higher overall task accuracy. In other words, whether stimulation occurred during or prior to task engagement shaped the observed outcomes. Like Ehrhardt et al (2021), this study reinforces that protocol differences (i.e., the timing of stimulation relative to task execution) can account for the variability in findings across the tDCS and multitasking enhancement literature.

Variations in stimulation location, as highlighted by Scheldrup and colleagues (2014), also contribute to the effects tDCS may have on dual-tasking. Anodal stimulation (2.0 mA) was administered for 30 min to either the left or right primary motor cortex (M1; C3, C4) or the left or right lateral frontal poles (F9, F10) as participants completed the gamified multitasking Space Fortress task (Mané & Donchin, 1989); in this task, participants navigate and manage a spaceship while responding to various challenges. Compared to sham, evaluation of the four sub-scores produced in the game revealed that right M1 stimulation improved the velocity and control sub-scores. In contrast, left frontal pole stimulation only improved control, and both left M1 and right lateral frontal pole stimulation had no significant effect. Of course, we must view these results in the context of the aforementioned studies stimulating the left DLPFC (F3), and the variety of other locations noted in Table 1. tDCS-induced dual-tasking outcomes, even within the same task, *highly* depend on electrode placement, and the large array of locations noted in the present review only contributes to outcome variability in the literature. This inconsistency drives the need for further research on the ideal current intensity, among other variables (e.g., stimulation duration, online vs. offline stimulation), when considering the benefits tDCS may have on dual-tasking.

**Table 1:** tDCS parameters and tasks of studies/experiments reviewed

*Study*	*Multitasking type*	*Task*	*Anode location(s)*	*Cathode location(s)*	*Frequency (mA)*	*Duration of stimulation (mins)*	*Control*	*Findings*
Abedanzadeh et al. (2021)	Dual-task (PRP)	Mixed SOA dual-task Stroop	Between F3 and C3	FP2	1.5	20 (Offline; 3 sessions: active or sham)	Sham–Stimulation discontinued after 30 s	Compared to sham, tDCS reduced RTs in both congruent and incongruent dual tasks, as well as across SOAs
Dubravac & Meier (2020)	Task-switching (switch costs)	Picture/word categorization task	P3; P6	P6; P3	1.0	~ 20 (Online; 1 session: active or sham)	Sham–stimulation discontinued after 30 s	Neither P3 nor P6 anodal stimulation significantly affected task-switching performance compared to sham
Ehrhardt et al. (2021)	Dual-task (non-PRP)	Visual-auditory dual-task; visual search transfer task	1 cm posterior to F3	Right supra-orbitofrontal cortex	0.7, 1.0, 2.0	20 (Online; 4 sessions: active or sham)	Sham–30 s linear ramp up and down	Anodal tDCS at 1.0 mA improved dual-task RT and both single and dual-task accuracy compared to sham. Stimulation at 0.7 mA improved single-task RT compared to sham, as well
Filmer et al. (2017)	Dual-task (non-PRP)	Trained and transfer single/dual tasks	1 cm posterior to F3	Right orbitofrontal region	0.7	13 (Online; 3 sessions: anodal, cathodal, or sham)	Sham–75 s of stimulation including 30 s ramp up and down	Anodal tDCS enhanced performance in untrained multitasking and visual search paradigms, but not a go/no-go task, with effects still evident in a 2-week follow-up
Filmer et al. (2013)	Dual-task (non-PRP)	Auditory/visual dual-task; auditory and visual single tasks	1 cm posterior to F3; FP2	FP2; 1 cm posterior to F3	0.7	9 (Offline; 3 sessions: 1 anodal, 1 cathodal, & 1 sham)	Sham–75 s of stimulation including 30 s ramp up and down	Compared to anodal or sham stimulation, cathodal tDCS significantly reduced dual-tasking costs
Gan et al. (2019)*	Dual-task (non-PRP)	Divided attention task	P3	P7, Pz, C3, O1	2.0	20 (Online; 4 sessions: 1 active HD-tDCS, 1 iTBS, 1 ProcTBS & 1 sham HD-tDCS)	Sham–30 s of ramping up to 1.0 mA and 30 s and down	No significant changes in divided attention dual three-back task performance were observed following HD-tDCS
Hafezi et al. (2024)	Dual-task (PRP)	SOA-varied Stroop Task	1 cm posterior to F3; Fp2	Fp2; 1 cm posterior to F3	1.0	20 (Offline; 4 sessions: anodal, cathodal, or sham)	Sham–30 s of stimulation at beginning and end of session	tDCS stimulation did not significantly reduce RT2 or PRP effects as compared to sham
Hemmerich et al. (2024)*	Dual-task (non-PRP)	ANTI-Vea Task	P4	CP2, CP4, PO4, PO8	1.5	~ 28 (Online; 1 session: active or sham)	Sham–30 s ramp up and down	HD-tDCS mitigated executive vigilance decrements in triple-load conditions, but not single or dual
Hsu et al. (2015)	Dual-task (non-PRP)	NeuroRacer	F3	Fp2	1.0	10 (Offline; 2 sessions, 1 active & 1 sham, or 2 sham)	Sham–30 s ramp up and down	Participants who received active tDCS in their first session saw reduced dual-tasking costs in their second session, only in multitasking conditions. Those that received 2 sham sessions or sham then active did not show dual-tasking performance changes
Kübler et al. (2025)	Dual-task (PRP)	Audiovisual dual-task paradigm	Between C3 and F3; Fp2	Fp2; Between C3 and F3	1.5	20 (Online; 2 sessions: Exp 1–1 anodal & 1 sham, Exp 2–1 cathodal & 1 sham)	Sham–30 s of stimulation including 10 s ramp up and 10 s ramp down	Anodal, but not cathodal, tDCS reduced RTs for order-switch trials. No effects on accuracy were observed for either protocol
Leite et al. (2020)	Task-switching (switch costs)	Parity/magnitude task	P3; P4	P4; P3	~ 2.0	30 (Online; 3 sessions: 1 anodal, 1 cathodal, & 1 sham)	Sham–15 s stimulation	Stimulation over P4, when compared to sham, increased switch cost RTs. No effects were seen on accuracy or of stimulation over P3
Leite et al. (2013)	Task-switching (switch costs)	Letter/digit naming task; vowel-consonant/parity task	F3; F4	F4; F3	2.0	30 (Online; 3 sessions: 1 anodal, 1 cathodal, & 1 sham)	Sham–15 s each of ramp up, plateau, and ramp down	Anodal tDCS over F3 reduced letter/digit switch costs in terms of RT, but increased vowel-consonant/parity RTs as compared to sham or cathodal stimulation
Leite et al. (2011)	Task-switching (switch costs)	Color/shape identification task	F3	FP2	1.0	15 (Online; 3 sessions: 1 anodal, 1 cathodal, & 1 sham)	Sham–15 s ramp up and down	Compared to sham or cathodal stimulation, anodal tDCS reduced switch costs
Mahesan et al. (2023)	Dual-task (PRP)	Audiovisual discrimination dual-task	F3	FP2	1.0	20 (Online; 2 sessions: 1 active & 1 sham)	Sham–30 s of stimulation including 10 s ramp up and 10 s ramp down	tDCS significantly reduced between-task interference in error rates when compared to sham stimulation. This was especially noted in high multitasking-demand conditions at short SOAs
Martin et al. (2014)	Dual-task (non-PRP)	Audiovisual dual N-back task	F3	Right upper arm	2.0	30 (Online and offline, 1 session each)	N/A	Online tDCS improved dual-tasking skill acquisition when compared to offline, while offline tDCS improved task accuracy when compared to online
Martin et al. (2013)	Dual-task (non-PRP)	Audiovisual dual N-back task	F3	Right upper arm	2.0	30 (Online; 10 sessions: active, sham + cognitive training (CT), or active + CT)	Sham–10 s ramp up down to 1.0 mA, 30 s 1.0 mA stimulation, and 10 s ramp down	Anodal tDCS paired with cognitive training contributed to improved task accuracy but not greater learning when compared to tDCS without cognitive training. In offline follow-ups, the tDCS + CT group did not significantly differ from sham
Nelson et al. (2019)^†^	Dual-task (non-PRP)	MATB	F3	Right upper arm	2.0	30 (Online; 1 session: active or sham)	Sham–15 s ramp up to 2.0 mA and 15 s ramp down	Compared to sham, anodal tDCS improved multitasking information throughput across workloads, as well as enhanced system monitoring and resource management subtask performance
Nelson et al. (2016)^†^	Dual-task (non-PRP)	MATB	F3	Right upper arm	2.0	30 (Online; 1 session: active or sham)	Sham–15 s ramp up to 2.0 mA and 15 s ramp down	Anodal tDCS significantly improved participants' overall information processing capability and multitasking throughput capacity compared to sham tDCS, especially in sustained attention and vigilance subtasks
Nikolin et al. (2019)*	Dual-task (non-PRP)	Divided attention task	F3; P3	F5, AF3, F1, FC3; P7, Pz, C3, O1	2.0	20 (Online; 1 session: active or sham)	Sham–30 s ramp up to 1.0 mA, 30 s ramp down	HD-tDCS did not significantly alter multitasking performance compared to sham
Perrotta et al., (2021, Exp. 2)	Dual-task (non-PRP)	Stroop/3-back dual-task	FP1	Crossing point between the lines connecting T4-Fz and F8-Cz	2.0	20 (Offline; 2 sessions: 1 active & 1 sham)	Sham–10 s ramp up, 7 s 2.0 mA stimulation, 10 s ramp down	Dual-tasking performance was not significantly changed by tDCS in either task
Prehn et al. (2021)	Task-switching (switch costs)	Letter-digit task	Halfway between F4 and C4; FP1	FP1; halfway between F4 and C4	1.0	20 (Online; 3 sessions: 1 anodal, 1 cathodal, & 1 sham)	Sham–10 s ramp up and 5 s ramp down after 30 s	Switch costs, but not task accuracy, were reduced by anodal tDCS. These effects were not task-specific
Rao et al. (2024)	Dual-task (non-PRP)	NASA-MATB-II; Dual audiovisual N-back task	F3	Fp2	1.0	15 (Online; 7 sessions: active or sham)	Sham–10 s ramp up and 5 s ramp down after 30 s	tDCS improved system monitoring and targeting performance as compared to sham but showed limited transfer effects for the untrained N-back task
Scheldrup et al. (2016)	Dual-task (non-PRP)	Warship Commander	F3; F4; contralateral upper arm	Contralateral upper arm; F3; F4	2.0	20 (Online; 1 session: anodal, cathodal, or sham)	Sham–ramp up to 2 mA then ramp down to 0.1 mA for remaining time	For low-performers, cathodal tDCS impaired multitasking performance on the monitoring subtask during stimulation and 24 h after compared to sham. Anodal stimulation, compared to sham, only impaired performance during the stimulation period
Scheldrup et al. (2014)	Dual-task (non-PRP)	Space Fortress	C3; C4; F9; F10	Contralateral arm	2.0	30 (Online; 1 session: active or sham)	Sham–ramp up to 2.0 mA then ramp down to 0.2 mA for remaining time	Compared to sham, anodal stimulation over C4 improved overall multitasking performance. C4 stimulation also improved control subtask performance compared to sham, and velocity subtask performance compared to C3, F9, or F10 stimulation. F9 stimulation improved speed subtask performance compared to sham, F10, or C3 stimulation
Sdoia et al. (2020)	Task-switching (switch costs)	Parity/magnitude task	F4; P4	F3; P3	1.5	Unspecified (Online; 3 sessions: 2 active & 1 sham)	Sham–ramp up and down for 45 s with 2 s of active stimulation	Anodal tDCS over F4 improved task-switching performance in both inhibited and non-inhibited conditions compared to sham. Anodal tDCS over P4, compared to sham, mitigated switch costs when returning to a previously inhibited task condition
Strobach et al. (2018)	Dual-task (PRP)	Audiovisual dual-task; auditory and visual single tasks	Halfway between F4 and C4; FP1	FP1; halfway between F4 and C4	1.0	20 (Online; 2 sessions: Exp 1–1 anodal & 1 sham, Exp 2–1 cathodal& 1 sham)	Sham–10 s ramp up, discontinued after 30 s	Under repeated task order and short SOA conditions, anodal tDCS reduced dual-tasking error rates when compared to sham. Cathodal tDCS, when compared to sham, increased error rates in different-order tasks. No effects of tDCS were observed for RT
Strobach et al. (2016)	Task-switching (switch and mixing costs)	Letter-digit task	Halfway between F3 and C3; FP2	FP2; halfway between F3 and C3	1.0	~ 20 (Online; 3 sessions: 1 anodal, 1 cathodal, & 1 sham)	Sham–10 s ramp up, discontinued after 30 s	Switch costs across sessions were not impacted by tDCS when compared to sham. When excluding practice effects, anodal tDCS increased mixing costs as compared to cathodal or sham
Strobach et al. (2015)	Dual-task (PRP)	Audiovisual dual-task; auditory and visual single tasks	Halfway between F3 and C3; FP2	FP2; halfway between F3 and C3	1.0	20 (Online; 2 sessions, Exp 1–1 anodal & 1 sham, Exp 2–1 cathodal & 1 sham)	Sham–10 s ramp up, discontinued after 30 s	Anodal tDCS, but not cathodal tDCS, improved performance in random-order dual tasks (decreased RTs and error rates) compared to sham
Tayeb and Lavidor (2016)	Task-switching (switch costs)	Parity/magnitude task	F3; F4	F4; F3	1.5	20 (Online; 3 sessions: anodal, cathodal, or sham)	Sham–30 s ramp up and down	Anodal stimulation over F3 AND over F4 reduced switch costs across sessions compared to sham
Wang et al. (2024)	Task-switching (switch and mixing costs)	Parity/magnitude task	P4	Left cheek	1.5	20 (Offline; 2 sessions: 1 active & 1 sham)	Sham–pseudo-stimulation for 30 s	tDCS did not significantly impact switch or mixing costs
Wang et al. (2022)	Task-switching (switch and mixing costs)	Parity/magnitude task	P3; P4	Right cheek; left cheek	1.5	20 (Offline; 1 session: active or sham)	Sham–pseudo-stimulation for 30 s	Anodal tDCS over P4 increased switch costs in predictable conditions compared to P3 or sham. No tDCS effects were seen in unpredictable conditions
Wang et al. (2021)	Task-switching (switch and mixing costs)	Parity/magnitude task; Parity/vowel-consonant task	F4	Left cheek	1.5	20 (Offline; 1 session: active or sham)	Sham–pseudo-stimulation for 30 s	Compared to sham, tDCS reduced switch costs in unpredictable parity/magnitude conditions and had no effect during predictable conditions. In the second task, tDCS increased switch cost in both unpredictable and predictable conditions
Wang et al. (2020)	Task-switching (switch and mixing costs)	Parity/magnitude task	F3; F4	Right cheek; left cheek	1.5	20 (Offline; 1 session: active or sham)	Sham–pseudo-stimulation for 30 s	Anodal stimulation over F4, when compared to sham and F3 stimulation, reduced switch costs in unpredictable task conditions. No significant effects were seen for predictable conditions or for mixing costs
Wards et al. (2023)	Dual-task (non-PRP)	Visual-auditory dual-task; visual search transfer task	1 cm posterior to F3; 1 cm posterior to F4	Right supra-orbitofrontal cortex; Left supra-orbitofrontal cortex	1.0; 2.0	13 (Online; 4 sessions: active or sham)	Sham–30 s linear ramp up and down; active stimulation control–1.0 mA stimulation with RSVP paradigm, 2.0 mA stimulation with multitasking training	tDCS did not significantly improve multitasking performance when compared to sham. However, 1.0 mA stimulation paired with multitasking training, regardless of laterality, did improve visual search speed in a transfer task when compared to sham and 2.0 mA stimulation
Zacharopoulos et al. (2020)*	Task-switching (switch costs)	Cued maintenance, distractor filtering, and stimulus updating task	F9; F4	Right shoulder; AF4, F6, F2, FC4	2.0	20 (Online; 3 sessions: 2 active & 1 sham)	Sham–60 s ramp up to 2.0 mA and 60 s ramp down	Task-switching performance was not significantly altered by tDCS

Tasks not pertaining to the scope of this review are not listed here. Asterisk (*) denotes the use of HD-tDCS. Obelus (^†^) denotes military-specific studies

Anode locations, as discussed in the review, are placed to target specific brain regions. We refer to them as follows: F3 or F4–left or right DLPFC, FC3 or FC4–left or right frontocentral region, F9 or F10–left or right lateral frontal poles, P3 or P4–left or right posterior parietal cortex (PPC), P6–right parieto-occipital region, C3 or C4–left or right primary motor cortices.

Additionally, individual differences have been noted to modulate dual-tasking at baseline (Jaeggi et al., 2007; Morgan et al., 2013), and may continue to play a role in these tasks when evaluating the enhancement capabilities of tDCS. Exploring the role of tDCS in modulating differing dual-tasking abilities, Scheldrup and colleagues (2016) divided their sample to receive either sham stimulation, anodal stimulation to the left (F3) or right (F4) DLPFC, or cathodal stimulation to the same locations. Stimulation was provided for 20 min at a current strength of 2.0 mA during the first of two days of testing. Warship Commander (St John et al., 2002), a multitasking videogame with concurrent monitoring and identification subtasks, was employed to assess dual-tasking abilities before, with, and without tDCS stimulation. In accordance with baseline target-identification RT and errors per mouse click, participants were clustered as low or high performers for analysis purposes. Low-performing participants who received *cathodal* stimulation experienced significant *increases* in errors committed, and similar results manifested for low-performers receiving right DLPFC (F4) anodal stimulation. Participants who initially performed well (i.e., high performers) did not experience significant performance effects as a result of any stimulation condition, and anodal left DLPFC (F3) stimulation yielded no effects, positive or negative. This study’s findings of cathodal stimulation stand in contrast with those of Filmer et al. (2013), who observed reduced reaction times in audiovisual dual-task conditions following 9 min of 0.7 mA of cathodal tDCS. These benefits identified by Filmer et al. (2013) were exclusive to both dual-tasking and cathodal tDCS, with no improvements produced in single-task conditions or with anodal tDCS. The marked differences in tDCS effects across participant-based (i.e., performance abilities) and stimulation-based (i.e., stimulation location, anodal vs. cathodal) factors are important to note.

Dual-task costs, as explored here, often appear to be reduced (or at least not increased) by anodal tDCS stimulation to the left DLPFC (F3). This is a finding determined across stimulation intensities, stimulation duration, and participant sample within the parameters of the present review. Given the widely-echoed concern over the heterogeneity of neurostimulation-based cognitive enhancement (Brunyé et al., 2024), the benefit provided by tDCS across conditions is a cautiously optimistic indication of the potential use of tDCS in applied settings for dual-tasking improvement.

### Psychological refractory period (PRP) effects

The second prominent way in which dual-tasking is evaluated is through the psychological refractory period (PRP) paradigm (Pashler, 1994; Welford, 1952), as it illustrates the cognitive limitations that may emerge when individuals attempt to process and respond to two stimuli presented in quick succession. In a dual-tasking PRP context, dual-task interference depends on the temporal overlap, typically studied in the order of milliseconds, between the two tasks rather than comparing single-task performance to dual-task performance. The interval between the onset of the first and second task, known as the stimulus onset asynchrony (SOA), significantly influences this dual-task interference. When the second task starts soon after the onset of the first task (i.e., shorter SOAs), performance on the second task declines compared to longer SOAs, and this decline in performance is called the PRP effect (Pashler, 1994). Though somewhat debated, the PRP effect is thought to occur because of information processing bottlenecks in which the response selection stages of both tasks must operate serially (Koch et al., 2018). We identified six articles where tDCS has been explored as a tool to target the PRP effect.

As was the case with dual-task costs, stimulation of the prefrontal cortex emerges as a common theme throughout the tDCS and PRP enhancement literature. For instance, Hafezi et al. (2024) applied either anodal or cathodal tDCS (1.0 mA for 20 min) over this area before participants completed an SOA-varied Stroop task while either fatigued or not fatigued. They identified the expected pattern of PRP effects such that shorter SOA trials produced greater performance impairments, but neither stimulation condition significantly reduced these effects relative to sham. In contrast, several other studies have reported improvements following DLPFC stimulation. In their 2023 study, Mahesan et al. found that anodal stimulation of this location proved promising at mitigating interference effects from the introduction of Task 2 (an auditory tone frequency identification task) during Task 1 (a visual, letter identification task) across short SOA conditions ( 1.0 mA for 20 min). These findings are echoed in a recent study by Kübler and colleagues (2025; 1.5 mA for 30 min), who assessed the dual-tasking performance effects of anodal and cathodal tDCS over the left DLPFC during varying task trial conditions and SOAs. The trial types (i.e., fixed-order, order-repetition, and order-switch) differed in whether participants followed a constant task sequence, repeated the previous task order, or intentionally changed the task order. While cathodal tDCS had no effect on performance, it was observed that, compared to sham, anodal tDCS contributed to shorter Task 2 response times in *order-switch* trials. Given that short SOAs (in the case of Mahesan et al., 2023) and varying task orders are associated with higher dual-task demands, the influence of tDCS over the left DLPFC (F3) observed in these studies aligns with a broader set of results suggesting dual-tasking enhancement via this region.

Similar findings were identified by Abedanzadeh and colleagues (2021), who administered 20 min of 1.5 mA of anodal tDCS at approximately FC3 in the 10–5 EEG system (i.e., the left IFJ). In their dual-task Stroop paradigm, participants receiving active tDCS showed significantly reduced reaction times to Stimulus 2 compared to sham across both short and long SOA conditions. This would indicate that, compared to sham, anodal stimulation of the left DLPFC effectively mitigated the PRP effect in the Stroop dual-task being used. Together, these findings suggest that anodal stimulation of left prefrontal cortex may alleviate PRP costs, particularly when dual-task demands are high. When applied over the *right* prefrontal cortex, improved performance in short SOA conditions is not lost; rather, using an audiovisual identification dual-task, Strobach et al. (2018) found reduced error rates with anodal tDCS (compared to sham) on trials with consistent task order and shorter SOAs (Experiment 1; 1.0 mA for 20 min). Viewed holistically, these studies highlight the role of the DLPFC in supporting dual-tasking and indicate that while effects are not uniformly observed, tDCS holds promise as a tool for enhancing aspects of cognitive control, depending on the target site and task characteristics like SOA length and trial order.

The interplay between electrode placement and task characteristics is further emphasized by Abedanzadeh et al. (2021) and Strobach et al., (2015, Experiment 1); in each study, significant interactions between active stimulation, task demands, and SOA were identified. Through these interactions, both studies demonstrated that anodal tDCS over the left DLPFC (F3) was most effective at reducing reaction times for the second task under short SOA and high-demand conditions. These short SOA scenarios are characterized by elevated cognitive load and pronounced task interference, where the central bottleneck in information processing becomes most apparent (Koch et al., 2018). This indicates that anodal tDCS may not only enhance the capacity for selective attention but may also improve the ability to resolve interference by facilitating adaptive cognitive control. These findings underscore the potential of tDCS as a tool for augmenting executive functions, particularly in challenging dual-task scenarios (i.e., short SOA conditions) where the demands on attention allocation and task coordination are maximal.

Emphasis must also be given to the fact that it is *anodal* tDCS that appears to induce dual-tasking improvements; *cathodal* tDCS, thought to inhibit neural activity, renders contrasting results. Bolstering the findings of Experiment 1, where anodal tDCS was shown to improve reaction time under high-demand conditions, Strobach et al. (2015, Experiment 2) employed 20 min of 1.0 mA *cathodal* tDCS over the left DLPFC. This resulted in impaired task-order preparation and decision-making during random-order audiovisual dual-task conditions. Participants exhibited slower RTs in different-order trials (i.e., trials when the task order changed) than in same-order trials after cathodal tDCS, an impairment that persisted even after the stimulation session. Importantly, though, PRP effects associated with shorter SOAs were not influenced by cathodal stimulation in this experiment, indicating that cathodal tDCS over the left IFJ may only *selectively* disrupt task-order control mechanisms in dual-task situations rather than the more general PRP effect. This selective disruption may be specific to cathodal tDCS, regardless of laterality; with cathodal tDCS administered to the right IFJ, compared to sham, error rates in different-order conditions increased (Strobach et al., 2018, Experiment 2). This finding effectively mirrors the findings of Strobach and colleagues’ 2015 study (Experiment 2) and may provide greater clarity on the conditions necessary to enhance dual-tasking with tDCS (i.e., anodal stimulation, rather than cathodal stimulation, over the prefrontal cortex is effective at improving dual-tasking). These results underscore the specificity of tDCS effects based on stimulation type and cortical region, highlighting that enhancements in dual-tasking are contingent upon *facilitating* rather than *inhibiting* neural activity. Consequently, future research and applications should prioritize anodal tDCS protocols targeting prefrontal regions to optimally target multitasking performance gains.

### Interim summary

Dual-tasking research highlights the role of tDCS, particularly anodal stimulation over the left DLPFC (F3), in mitigating dual-task costs. Studies by Hsu et al. (2015) and Filmer et al. (2017) demonstrate that anodal tDCS targeting DLPFC can enhance multitasking performance and produce both immediate and long-term cognitive benefits. Pairing anodal tDCS of this location with multitasking training, as noted by Martin et al. (2013), Rao et al. (2024), and Wards et al. (2023), also shows promise in improving cognitive outcomes. However, variations in stimulation intensity (e.g., 0.7 mA to 2.0 mA), electrode location, stimulation timing, and individual baseline performance can modulate these effects, as shown by Ehrhardt et al. (2021), Martin et al. (2014), and Scheldrup et al. (2016). While stimulation over the left DLPFC (F3) appears most consistently beneficial, with notable exceptions (e.g., Hafezi et al., 2024), other cortical sites (e.g., right M1 or left lateral frontal pole) show mixed effects depending on task demands, highlighting the need for further research on optimal stimulation parameters.

Similarly, PRPs show promise for improvement with anodal tDCS over the left DLPFC. Studies by Mahesan et al. (2023) and Abedanzadeh et al. (2021) demonstrate reduced reaction times and improved performance in high-demand, short SOA conditions, where task interference is most pronounced. In contrast, cathodal tDCS tends to impair task-order control, as seen in findings by Strobach et al., (2015, 2018), emphasizing that enhancements in dual-tasking and PRP outcomes may be limited to anodal stimulation. These results underscore the DLPFC’s key role in one type of multitasking and highlight the potential for tDCS to enhance cognitive control in demanding operational contexts, such as those encountered in military training and operations.

## High-definition tDCS and multitasking

Newer to neuromodulation research is high-definition transcranial direct current stimulation (HD-tDCS), an advancement to improve the existing tDCS technique. As illustrated by the variety of stimulation intensities and duration outlined above, there exists an issue of heterogeneity in the application of tDCS for the purposes of multitasking enhancement. It proves challenging to recommend a technique for applied purposes with so many inconsistencies, and those listed do not cover the extent of variations in the literature; studies also vary in the size of the tDCS electrodes (e.g., 5 × 5 cm^2^, 5 × 7 cm^2^, 4 × 4cm^2^, etc.). Additionally, the large size of these electrodes means that stimulation is broadly, rather than focally, delivered.

HD-tDCS serves as a potential solution to these complications (Villamar et al., 2013). This technique commonly uses a 4 × 1 ring configuration over a region of interest, in which a central electrode that determines polarity (anodal, cathodal) is surrounded by four return electrodes to complete the electrical circuit. As a result, the area receiving stimulation is suggested to be more focal than that during conventional tDCS due to the return electrodes constraining the diffusion of electrical current to other cortical regions (Alam et al., 2016; Datta et al., 2009). While considerations for stimulation intensity, configuration, and ring diameter must be given (Alam et al., 2016; Datta et al., 2008), HD-tDCS improves the specificity with which the conclusions drawn from tDCS can be given to the targeted cortical area. There are also indications that the effects of HD-tDCS may last longer following stimulation than those of conventional tDCS (Kuo et al., 2013). Like conventional tDCS, HD-tDCS is safe and tolerable for both clinical and healthy populations (Borckardt et al., 2012; Reckow et al., 2018; Richardson et al., 2015; Turski et al., 2017), perhaps even more tolerable than conventional tDCS (Gbadeyan et al., 2016). Both researchers and participants, then, stand to benefit from HD-tDCS.

### HD-tDCS & task-switching

Only one paper was identified that used HD-tDCS to enhance task-switching performance. In addition to their conventional tDCS montage over the left lateral frontal pole (F9), Zacharopoulos et al. (2020) employed an HD-tDCS montage targeting the right DLPFC (F4) to explore its effects on task-switch costs. No significant impact on task accuracy or response time was observed (i.e., switch cost was not affected by 20 min of 2.0 mA anodal HD-tDCS). However, their Supplemental Material introduces a nonsignificant correlation between task-switching accuracy outcomes for conventional tDCS over the left lateral frontal pole and HD-tDCS targeting the right DLPFC. This would imply that the two differentially affected task-switching, introducing the interesting question of *how* conventional and HD-tDCS differ in their impacts on multitasking. Future studies should systematically compare these two stimulation modalities across a range of tasks and conditions to clarify their relative advantages improving task-switching performance.

### HD-tDCS & dual-tasking

Out of the 22 articles identified in this review as having investigated dual-tasking, just three employed HD-tDCS. Exploring how this technique has been used in the dual-task enhancement literature provides insight into the future of neurostimulation for multitasking improvement; however, as suggested by the following studies, it may be best to stick to the status quo (i.e., conventional tDCS) when considering the use of this technology to improve dual-task abilities.

For the most part, anodal HD-tDCS has rendered null findings regarding enhancement of dual-tasking. Hemmerich and colleagues (2024) demonstrated no improvement in single- or dual-tasking with 28 min of 1.5 mA HD-tDCS targeting the right PPC (P4), although a mitigated executive vigilance decrement was observed in triple-task conditions. While this does not bode well for dual-task enhancement, it suggests that HD-tDCS benefits may be contingent on sufficiently high cognitive demands, allowing the stimulation to engage task-relevant processes effectively without ceiling effects or overstimulation. Similarly, both Gan et al. (2019) and Nikolin et al. (2019) reported no improvements in a divided attention dual three-back task following HD-tDCS, with 20 min of 2.0 mA stimulation applied to the left PPC (P3) in both studies; Nikolin et al. (2019) also included participants who received stimulation at the left DLPFC (F3), which also showed nonsignificant effects. Considering what this review has noted in relation to the effects of anodal tDCS over the left DLPFC, it is curious that dual-tasking improvements have been observed with conventional tDCS on the left DLPFC (F3) but not with HD-tDCS. As with task-switching, this raises questions about the differing mechanisms and efficacy between the two techniques and should serve to inspire further inquiries into the implications of this discrepancy when studying multitasking enhancement.

Despite these results, HD-tDCS should not be wholly written off in the multitasking enhancement literature. It is unclear as to why HD-tDCS results in multitasking appear lackluster, but increased focality, marketed as a benefit of HD-tDCS, may contribute to this trend. Due to the decreased distance between the target and return electrodes, HD-tDCS is more prone to shunting effects (i.e., the current dissipates across the scalp leading to less current penetrating the skull and CSF to the brain tissue; Bikson et al., 2010; Miranda et al., 2006). This is further complicated by the use of smaller electrodes in many HD-tDCS setups, which may increase the shunting effect (Wagner et al., 2007). The diffuse nature of conventional tDCS, unlike HD-tDCS, may allow for a broader span of regions associated with multitasking to be stimulated with a lower risk of shunting, producing the variety of results examined in this review. Balancing focality and current penetration is delicate and must be thoroughly investigated. As a result, systematic comparisons of conventional and HD-tDCS must be conducted when considering their potential use in multitasking enhancement interventions.

Additionally, just four studies (n_Task-Switching_ = 1; n_Dual-Tasking_ = 3) are discussed in this section on HD-tDCS, relative to the 31 conventional tDCS studies that have been examined thus far in the present review; additional research on HD-tDCS’s respective effects on dual-tasking and task-switching is critical before any final determination on its efficacy can be made. As greater focus is given to neuromodulation at large, it is our hope that HD-tDCS is employed in future studies on multitasking to fully establish its potential in this area of research.

## tDCS in military multitasking

Use of tDCS in a military context requires the consideration that individuals, stimulation protocols, and tasks all may impact the enhancement capacity of this technology. This same context requires recognition that single-measure evaluation of the effects of tDCS on task-switching and dual-tasking will likely not capture the full picture. Complex interactions exist between stimulation parameters, participant samples, and task requirements, and, unless a complex overview of results accounting for these variables is rendered, we must view tDCS enhancement of multitasking with a critical eye. With that said, we identified two studies assessing the ability for tDCS to modulate military dual-tasking.

Both identified studies used the same task: the Multi-Attribute Task Battery (MATB). The MATB is a NASA-created multitasking environment consisting of four independent subtasks (system monitoring, communication, targeting, and resource management) that must be simultaneously monitored and responded to (Comstock & Arnegard, 1992); this task has been used in numerous studies on military multitasking, and may even *predict* real-world military multitasking ability (Hilla et al., 2024). Contributing to this body of research, Nelson and colleagues () recruited participants from Ohio’s Wright-Patterson Air Force Base to test the effects of anodal tDCS over the left DLPFC (F3) during the Air Force Multi-Attribute Task Battery (AF-MATB; Miller et al., 2014). Both studies administered anodal stimulation for 30 min at a current strength of 2.0 mA. It is also important to note that multitasking performance in these studies was operationalized with throughput capacity, a measure of the efficiency with which participants process information, rather than the previously discussed measures of multitasking performance. Nelson et al. (2016) revealed subtask-specific enhancements of multitasking ability following left DLPFC anodal tDCS, with significantly increased throughput percentages displayed for the system monitoring and resource management subtasks.

These findings were bolstered by Nelson and colleagues (2019), this time supported by eye-tracking evidence suggesting that these specific subtasks were subject to fewer fixations. Enhanced multitasking performance following anodal tDCS to the DLPFC, specifically for system monitoring and resource management, is particularly interesting. Linked to sustained attention and vigilance, anodal tDCS increased throughput capacity for these subtasks, suggesting broader implications for improving attentional (and multitasking) abilities with anodal tDCS to DLPFC. System monitoring and resource management are also essential military duties. As such, an established protocol for enhancing the ability to attend to and simultaneously engage with these tasks could prove valuable for military personnel.

Despite the limited number of studies specifically examining tDCS effects on military-relevant multitasking performance, there are several potential military applications for techniques like tDCS as they emerge in the scientific literature. Below, we review five potential military applications that are likely to have a high impact on training, readiness, situational awareness, and task effectiveness:

*Skill Acquisition During Training*. Military personnel are required to master complex cognitive and motor tasks under time constraints and pressure. During skill acquisition, task-switching and dual-tasking are often necessary as trainees alternate between learning objectives (e.g., weapons operation, vehicle controls) while attending to instructions or situational cues. Anodal tDCS targeting the DLPFC, particularly when paired with a multitasking training regimen, could accelerate learning by enhancing working memory and cognitive flexibility, allowing trainees to process and integrate new information more efficiently. For example, improved task-switching abilities during training for vehicle operation could enable faster transitions between navigation, target identification, and communications tasks.

*Seated Analyst Roles*. Intelligence analysts, air traffic controllers, and cybersecurity personnel often perform tasks that require sustained attention, rapid task-switching, and prolonged vigilance. Analysts monitor multiple screens, identify critical information, and respond to dynamic changes, all of which place high cognitive demands on executive control, working memory, and attention. Anodal tDCS targeting the left DLPFC could improve dual-tasking and enhance vigilance (Filmer et al., 2017; Hemmerich et al., 2024; Hsu et al., 2015), bolstering analysts’ ability to transition between tasks (e.g., processing signals intelligence and composing situation reports or communicating) without performance degradation. Additionally, the observed effects of tDCS on reduced response times without compromising accuracy (Leite et al., 2011; Prehn et al., 2021) could enable analysts to act on critical information more swiftly, reducing decision-making delays in time-sensitive scenarios.

*Drone Operator Tasks.* Drone operators are tasked with simultaneously piloting unmanned aerial vehicles, monitoring video feeds, and communicating with team members. These roles are expected to expand in future operations, as human–machine integrated formations are supported by robotic and autonomous systems, requiring both dual-tasking (e.g., operating controls while interpreting visual data) and task-switching (e.g., shifting focus between multiple drones or information sources). Evidence suggests that tDCS applied to the right DLPFC may improve cognitive control during unpredictable tasks (Wang et al., 2020, 2021), enhancing operators’ ability to manage sudden task demands, such as identifying and responding to emerging threats. This could lead to fewer errors, improved response times, and enhanced situational awareness.

*Infantry Operations*. Infantry personnel frequently face overlapping cognitive and motor task demands, such as maintaining situational awareness, monitoring communications, and engaging targets, all while operating in unpredictable and high-stress environments. Multitasking under these conditions could increase dual-tasking costs and errors, particularly when quick transitions between simultaneous tasks are required. Targeted tDCS interventions, especially when paired with cognitive training protocols, could enhance cognitive flexibility, allowing infantry personnel to adapt more quickly to shifting priorities, such as when scanning for enemy movement while coordinating plans with teammates.

*Pilot and Aircrew Roles*. Pilots and aircrew must balance multiple concurrent demands, including navigation, team communication, and system monitoring. Effectively managing switch costs and sustaining readiness in mixed-task environments is critical for maintaining operational efficiency. Results from studies using the MATB suggest that tDCS over the DLPFC could enhance system monitoring and resource management tasks (Nelson et al., 2016, 2019), which are central to pilot performance. Enhanced vigilance and multitasking efficiency may lead to fewer errors of omission (e.g., missed warnings) and improved situational awareness during extended flight operations.

## Limitations

Several limitations of the present review must be acknowledged. Critically, only 35 articles met all inclusion criteria and were ultimately incorporated into our exploration of how tDCS affects cognitive multitasking; these 35 papers were then divided to better facilitate comparisons between those with similar multitasking metrics. The limited overall number of studies, as well as their breakdown into thematic subsections, certainly may restrict the robustness of the conclusions drawn in this review. Nonetheless, we elected to maintain these subsections; we found this structure valuable for underscoring the nuanced effects of tDCS across components of multitasking as it is commonly measured, potentially mapping onto the distinct neural substrates that support different aspects of multitasking performance. Dedicated, network-level syntheses of these effects would, we believe, be a valuable direction for future work.

As noted throughout this review, there was substantial variability in stimulation parameters, complicating efforts to synthesize findings and provide recommendations regarding tDCS and military multitasking. Variations were observed in electrode placements, (e.g., F3, P4, C3, etc.), current intensity (ranging from 0.7 mA to 2.0 mA), stimulation duration (ranging from 9 to 30 min), and the use of online versus offline stimulation protocols. Differences in cognitive tasks, electrode sizes, sham protocols, and the tDCS devices used were also noted across the included studies. With just 35 articles discussed in this review, these methodological discrepancies create challenges in determining whether multitasking outcomes are attributable to the specific stimulation parameters in each study, or more broadly to tDCS as an intervention. Greater standardization of stimulation protocols in future studies on tDCS and multitasking performance, including in military settings (See van der Groen et al., 2025), is therefore, encouraged.

Other limitations inherent to tDCS as a methodological approach should also be noted. The nature of tDCS is modulatory, with electric current only making it more or less likely for an action potential to occur (rather than inducing neuronal activity, such as with TMS). Further, tDCS has a relatively low spatial resolution, limiting our ability to understand how such modulation impacts specific neural targets (Priori et al., 2009). As discussed in *High-Definition tDCS and Multitasking*, advancements made in tDCS technology to address this limitation (i.e., HD-tDCS) are also subject to their own complications, such as increased current shunting across the scalp (Bikson et al., 2010; Miranda et al., 2006; Wagner et al., 2007). Nonetheless, ongoing efforts to understand and improve the focality of tDCS, including the reduction of shunting (e.g., Faria et al., 2011; Neri et al., 2020; Niemann et al., 2024), are positive indications for the spatial resolution of future tDCS studies. The same optimism for tDCS may be applied to other concerns, such as individual variability in effectiveness. Pertinent to many neuromodulation techniques, including tDCS, differences between individuals have the ability to influence neuromodulation-induced outcomes (Berryhill & Martin, 2018; Krause & Cohen Kadosh, 2014), complicating the ability to generalize conclusions. As the body of research on tDCS grows, the increased focus on these subject-level factors (e.g., Guerra et al., 2020; Vergallito et al., 2022) may contribute to stimulation protocol standardization and greater understanding of tDCS as a viable technique to alter cognitive function, including multitasking abilities.

Finally, it must also be acknowledged that there is a lack of certainty regarding the long-term effects of tDCS on multitasking performance. The majority of studies included in this review assessed outcomes yielded during stimulation or shortly after stimulation, with a notable few incorporating follow-up assessments beyond the immediate post-stimulation window. As such, it is unclear whether any observed changes in multitasking performance reflect short-lived shifts or more durable changes in cognitive functioning. However, observations of other cognitive abilities, such as working memory, have demonstrated that tDCS can yield benefits that persist for weeks or even months after stimulation (e.g., Au et al., 2016; Katz et al., 2017), suggesting that more sustained effects are possible under certain conditions. These findings raise important questions about the mechanisms and time course of tDCS-induced plasticity, as well as the extent to which they can be harnessed safely in multitasking contexts. In this regard, it is reassuring that the duration of neuroplastic after-effects following a single session of tDCS, though subject- and protocol-dependent, are not considered to pose any risk. Typically observed to last approximately 60–120 min (Jamil et al., 2017; Monte-Silva et al., 2013; Nitsche & Paulus, 2001; Stagg et al., 2018), tDCS’s after-effect temporal profile aligns with those of other single-session NIBS techniques, including rTMS (Ziemann et al., 2008), tRNS (Terney et al., 2008) and tACS (Agboada et al., 2025; Kasten et al., 2016). Future multitasking research would benefit from incorporating long-term follow-ups to better understand the persistence and functional significance of tDCS-induced effects. As it stands, though, current evidence supports the short- and long-term safety of the technique, as well as its potential to elicit acute enhancements in cognitive performance.

## Conclusions

Transcranial direct current stimulation has been studied many times for its ability to enhance multitasking performance, and as seen in this review, its success is mixed; while promising findings have been identified for task-switching and dual-tasking, nonsignificant effects are also commonplace throughout the literature. When assessing the future of tDCS in the modulation of military multitasking, though, this technology must still be considered. The present review identified just 35 studies expressly testing the effects of tDCS on multitasking, and these papers vary considerably in the stimulation parameters employed and tasks used. Variables such as stimulation intensity, electrode placement, session duration, and task requirements play critical roles in shaping outcomes, and further investigation is required to address the inconsistencies between studies in this respect. Multitasking is a multi-faceted cognitive skill, and clear parameters for tDCS are a necessary first step when considering the enhancement of this skill via neuromodulation.

Moreover, just four studies examined the effects of HD-tDCS on multitasking, and none assessed this technology in regard to military populations. This highlights a significant gap in the literature, as HD-tDCS, with its enhanced focality and precision compared to conventional tDCS, may offer unique benefits for improving multitasking in demanding, high-stakes environments like those encountered in military operations. The more-frequent use of applied tasks like MATB should also be considered for future studies; these tasks simulate the complex, dynamic scenarios that closely mirror real-world demands faced by military personnel and average citizens, alike. Incorporating MATB or similar paradigms could provide more ecologically valid insights into the potential of both conventional and HD-tDCS to enhance multitasking performance under stress. While the potential applications of tDCS for military multitasking are intriguing, the current evidence base is insufficient to recommend its widespread adoption in training or operational environments. The variability in outcomes, combined with the limited number of studies addressing military-relevant tasks, underscores the need for further research to establish reliable stimulation protocols and the nature of task-specific benefits.

As the evidence base grows, tDCS may hold promise for enhancing multitasking across diverse military roles, from improving skill acquisition during training to reducing cognitive overload in analyst, drone operator, infantry, and aircrew tasks. These advancements could ultimately improve readiness, situational awareness, and task effectiveness in high-stakes environments, provided future research addresses the critical gaps and challenges identified in the current literature. Addressing these gaps will not only provide critical insights into the practical utility of tDCS for military application, but, importantly, advance our understanding of tDCS as a tool for cognitive enhancement and continue to elucidate the mechanisms driving any such effects.

## Data Availability

Not applicable.

## Appendix A

### Modified Downs and Black checklist for assessment of methodological quality.

This version of the Downs and Black Checklist (1998) was modified to better fit nonclinical tDCS studies. Article quality was determined by the proportion of article score to maximum possible score. In this form of the checklist, item 24 is modified to whether a power analysis was performed (MacLehose et al., 2000). Additionally, item 8 was modified such that studies that both measured *and* reported adverse effects pertaining to tDCS received a “Yes,” while those that measured adverse effects but did not report their findings received a “Partially.” This modification was modeled after the structure of item 5. In total, articles could report a maximum of 25 points.

**Table:**

Item	Criteria	Answers
Reporting		
1	*Is the hypothesis/aim/objective of the study clearly described?*	Yes = 1 No = 0
2	*Are the main outcomes to be measured clearly described in the Introduction or Methods section?* If the main outcomes are first mentioned in the Results section, the question should be answered no	Yes = 1 No = 0
3	*Are the characteristics of the patients included in the study clearly described?* In cohort studies and trials, inclusion and/or exclusion criteria should be given. In case–control studies, a case definition and the source for controls should be given	Yes = 1 No = 0
4	*Are the interventions of interest clearly described?* Treatments and placebo (where relevant) that are to be compared should be clearly described	Yes = 1 No = 0
5	*Are the distributions of principal confounders in each group of subjects to be compared clearly described?*	Yes = 2 Partially = 1 No = 0
6	*Are the main findings of the study clearly described?* Simple outcome data (including denominators and numerators) should be reported for all major findings so that the reader can check the major analyses and conclusions. (This question does not cover statistical tests which are considered below)	Yes = 1 No = 0
7	*Does the study provide estimates of the random variability in the data for the main outcomes?* In non-normally distributed data, the interquartile range of results should be reported. In normally distributed data, the standard error, standard deviation, or confidence intervals should be reported. If the distribution of the data is not described, it must be assumed that the estimates used were appropriate and the question should be answered yes	Yes = 1 No = 0
8	*Have all important adverse events that may be a consequence of the intervention been measured AND reported?* This should be answered yes if the study demonstrates that there was a comprehensive attempt to measure adverse events. (A list of possible adverse events is provided)	Yes = 2 Partially = 1 No = 0
9	*Have the characteristics of patients lost to follow-up been described?* This should be answered yes where there were no losses to follow-up or where losses to follow-up were so small that findings would be unaffected by their inclusion. This should be answered “no” where a study does not report the number of patients lost to follow-up	Yes = 1 No = 0
10	*Have actual probability values been reported (e.g., 0.035 rather than* < *0.05) for the main outcomes except where the probability value is less than 0.001?*	Yes = 1 No = 0
Internal validity–bias		
11	*Was an attempt made to blind study subjects to the intervention they have received?* For studies where the patients would have no way of knowing which intervention they received, this should be answered yes	Yes = 1 No = 0 Unable to determine = 0
12	*Was an attempt made to blind those measuring the main outcomes of the intervention?*	Yes = 1 No = 0 Unable to determine = 0
13	*If any of the results of the study were based on “data dredging,” was this made clear?* Any analyses that had not been planned at the outset of the study should be clearly indicated. If no retrospective unplanned subgroup analyses were reported, then answer yes	Yes = 1 No = 0 Unable to determine = 0
14	*In trials and cohort studies, do the analyses adjust for different lengths of follow-up of patients, or in case–control studies, is the time period between the intervention and outcome the same for cases and controls?* Where follow-up was the same for all study patients the answer should be yes. If different lengths of follow-up were adjusted for by, for example, survival analysis the answer should be yes. Studies where differences in follow-up are ignored should be answered no	Yes = 1 No = 0 Unable to determine = 0
15	*Were the statistical tests used to assess the main outcomes appropriate?* The statistical techniques used must be appropriate to the data. For example, nonparametric methods should be used for small sample sizes. Where little statistical analysis has been undertaken but where there is no evidence of bias, the question should be answered yes. If the distribution of the data (normal or not) is not described it must be assumed that the estimates used were appropriate and the question should be answered yes	Yes = 1 No = 0 Unable to determine = 0
16	*Was compliance with the intervention/s reliable?* Where there was noncompliance with the allocated treatment or where there was contamination of one group, the question should be answered no. For studies where the effect of any misclassification was likely to bias any association to the null, the question should be answered yes	Yes = 1 No = 0 Unable to determine = 0
17	*Were the main outcome measures used accurate (valid and reliable)?* For studies where the outcome measures are clearly described, the question should be answered yes. For studies which refer to other work or that demonstrate the outcome measures are accurate, the question should be answered as yes	Yes = 1 No = 0 Unable to determine = 0
Internal validity–confounding (selection bias)		
18	*Were the patients in different intervention groups (trials and cohort studies) or were the cases and controls (case–control studies) recruited from the same population?* For example, patients for all comparison groups should be selected from the same hospital. The question should be answered unable to determine for cohort and case–control studies where there is no information concerning the source of patients included in the study	Yes = 1 No = 0 Unable to determine = 0
19	*Were study subjects in different intervention groups (trials and cohort studies) or were the cases and controls (case–control studies) recruited over the same period of time?* For a study which does not specify the time period over which patients were recruited, the question should be answered as unable to determine	Yes = 1 No = 0 Unable to determine = 0
20	*Were study subjects randomized to intervention groups?* Studies which state that subjects were randomized should be answered yes except where method of randomization would not ensure random allocation. For example, alternate allocation would score no because it is predictable	Yes = 1 No = 0 Unable to determine = 0
21	*Was there adequate adjustment for confounding in the analyses from which the main findings were drawn?* This question should be answered no for trials if: the main conclusions of the study were based on analyses of treatment rather than intention to treat; the distribution of known confounders in the different treatment groups was not described; or the distribution of known confounders differed between the treatment groups but was not taken into account in the analyses. In non-randomized studies if the effect of the main confounders was not investigated or confounding was demonstrated but no adjustment was made in the final analyses the question should be answered as no	Yes = 1 No = 0 Unable to determine = 0
22	*Were losses of patients to follow-up taken into account?* If the numbers of patients lost to follow-up are not reported, the question should be answered as unable to determine. If the proportion lost to follow-up was too small to affect the main findings, the question should be answered yes	Yes = 1 No = 0 Unable to determine = 0
*Power*		
24	*Was a power analysis performed?*	Yes = 1 No = 0 Unable to determine = 0

Three questions from the checklist were removed from this version due to their lack of relevance to nonclinical tDCS studies. They were: 1) “*Were the subjects asked to participate in the study representative of the entire population from which they were recruited?*” 2) “*Were those subjects who were prepared to participate representative of the entire population from which they were recruited?*” and 3) “*Were the staff, places, and facilities where the patients were treated, representative of the treatment the majority of patients receive?*” Due to redundancy with items 11 and 12, we also removed the question “*Was the randomized intervention assignment concealed from both patients and health care staff until recruitment was complete and irrevocable?*”.

## Abbreviations

AF-MATB: Air Force Multi-Attribute Task Battery
DLPFC: Dorsolateral prefrontal cortex
EEG: Electroencephalogram
HD-tDCS: High-definition transcranial direct current stimulation
IFJ: Inferior frontal junction
mA: Milliamps
MATB: Multi-Attribute Task Battery
NIBS: Noninvasive brain stimulation
PRP: Psychological refractory period
RT: Response time
SOA: Stimulus onset asynchrony
tDCS: Transcranial direct current stimulation
